# Archiving time series sewage samples as biological records of built environments

**DOI:** 10.1186/s12879-021-06268-4

**Published:** 2021-06-24

**Authors:** David S. Thaler, Thomas P. Sakmar

**Affiliations:** 1 Biozentrum, University of Basel, CH-4056 Basel, Switzerland; 2 Program for the Human Environment, Rockefeller University, New York, NY USA; 3 Laboratory of Chemical Biology and Signal Transduction, Rockefeller University, New York, NY USA

**Keywords:** SARS-CoV-2, Covid-19, Virus, Sewage, Wastewater, Sample archiving, Historical epidemiology, Public health, Biological diaries

## Abstract

This commentary encourages the regular archiving of nucleic-acid-stabilized serial samples of wastewaters and/or sewage. Stabilized samples would facilitate retrospective reconstitution of built environments’ biological fluids. Biological time capsules would allow retrospective searches for nucleic acids from viruses such as SARS-CoV-2. Current resources for testing need not be diverted if samples are saved in case they become important in the future. Systematic storage would facilitate investigation into the origin and prevalence of viruses and other agents. Comparison of prevalence data from individual and clinical samplings with community wastewater would allow valuable comparison, contrast and correlation among different testing modalities. Current interest is focused on SARS-CoV-2, but archived samples could become valuable in many contexts including surveys for other infectious and chemical agents whose identity is not currently known. Archived time series of wastewater will take their place alongside other biological repositories and records including those from medical facilities, museums, eDNA, living cell and tissue collections. Together these will prove invaluable records of the evolving Anthropocene.

## Background

The purpose of this commentary is to encourage the regular archiving of nucleic-acid-stabilized serial samples of untreated waste water and sewage from multiple systems and subsystems of our built environment. There is an important literature on preserving wastewater samples [1, 2] for chemical analysis. Nucleic acid-stabilized samples could preserve materials allowing future insights into the biological fluids of built environments, including evidence of viruses such as SARS-CoV-2. Imagine if we had a series of samples before and during the arrival of SARS-CoV-2 into a city, apartment block, or cruise ship. Retrospective analysis would enrich the investigation of questions about the origin and prevalence of the virus, which would be answerable in a more objective way than other methods allow.

There is a significant and rapidly growing literature on SARS-CoV-2 virus detection in sewage, including tracing the virus to specific buildings; surely more use of sewage for “real time” analysis will come [3–11]. It seems too late for archived samples to allow tracing the first, or second waves in many locales but the need to monitor infection will certainly continue. Retrospective information will inform the analysis and understanding of public health measures, for example, how restrictions influenced subsequent viral load in a community. Testing of individuals is vulnerable to different sampling biases than averaging the collective biological fluid of a larger community. Comparison of prevalence data from individual and clinical samplings with community sewage would allow valuable correlation that may help dispel critiques such as “More testing results in more positive cases.” [12].

Biological diaries, archives, and culture collections are invaluable in contexts including medical repositories, museums, collections of living and revivable tissues and cells in biobanks, seed banks of biodiversity, and many other contexts [13, 14]. Biological diaries of built environments stored and analyzed in similar ways to analogous time-series repositories from less-human-centered environments could synergistically deepen understanding of the dynamic and evolving Anthropocene. There is a recognized need for better biobanks from complex microbial systems [15].

## Main text

Historical samples have proven important in tracing the origin and spread of infectious diseases in previous instances. However, in the cases of which we are aware, biological materials were originally collected for other purposes. Molecular methods of analysis used, to study the origin of HIV, for example, were not even dreamed of at the time samples were taken [16]. Molecular reexamination of medical samples from the 1918–1919 flu pandemic led to the interpretation, nearly 100 years later, that much mortality was due to secondary bacterial pneumonia [17]. The storage and availability of samples ready for nucleic acid sequence studies has often been haphazard, perhaps because there has been insufficient articulation of their value.

There are many contexts for which a properly preserved time series of biological samples of built environments are potentially of inestimable value. Archeologists and physical anthropologists make good use of the information obtained from ancient latrines, valuable biological time capsules that were not deliberately created [18–22]. Museums and other collections and repositories contain materials that can be explored again and again as new methods are developed and the value of information obtainable from these samples is realized.

Environmental DNA (eDNA) is increasingly recognized and used for species assays in aquatic systems to supplement or partially replace trawl or other macroscopic methods of census [23, 24]. Methods are being refined to make archiving and subsequent information extraction more efficient, but approaches for reliable archiving of microbiome and other biological samples, indefinitely, at ambient temperature, are already well established [25, 26]. The key point is that it is not even necessary to isolate nucleic acids right away.

Leonardo Da Vinci conceived of Earth and also architectural entities such as cities as akin to living organisms [27]. The consideration of houses and cities as bodies was an important part of new and influential conceptualizations in public health that began in late nineteenth-century England and continue to influence architecture and urban design [28, 29]. Cross-fertilization between innovations of precision medicine as applied to individual patient care and “precision public health” are especially relevant in the context of infectious disease (and in a hopeful future, of infectious health [30, 31]). Biological archiving of samples has promise in both contexts of individuals and of environments, be they built, natural, or any combination.

Analytic methods are likely to improve over the coming years and to increase the information that can be obtained from archived samples. The most exciting developments might be unanticipated, but some possibilities for innovation are outlined below.

### Contexts and scenarios where nucleic acid stabilized wastewater samples could help

Retroactive analysis of wastewater samples has the potential to inform epidemiology, kinetics in closed and semi-closed systems, and the understanding of microbial evolution in built environments. Sewage samples archived for other purposes and stored at -20 °C have been retroactively assayed for SARS-CoV-2 and found positive [32]. When did a virus first begin to spread in a city, or on a particular cruise ship, aircraft carrier [33], or future interplanetary expedition? How quickly did the public titer of virus change? Does public virus titer follow a logistic curve or other kinetic as predicted by various models? How did various public health measures such as lockdowns, quarantines, mask wearing, affect the public pool of virus?

Consider the controversy concerning the origin of SARS-CoV-2 [34–38]. Archived samples of building sewage from relevant laboratories, markets, and neighborhoods would be of inestimable value in providing more threads to pull on in order to better understand what has happened. One thing is certain: This SARS-CoV-2 outbreak will not be the last pandemic, even as it is not the first whose origin has been controversial (e.g. [39–41]).

The number of positive cases determined from individual tests has several uncertainties. How are people to be tested chosen? The kinetics of viral appearance and disappearance in individuals are uncertain variables. The shared fluid of built environments is a complementary sampling strategy that pools all the individuals, and with statistical qualifications has been found to agree reasonably well with individual sampling from the community [42]. Depending on how sampling is done, sources can be pinpointed in both space and time, for example to branch points in the collection system.

PCR and rtPCR based methods for virus detection now largely depend on having the target sequence in hand. Non-targeted detection methods are becoming more sophisticated, allowing detection of genetic elements and agents that have not been previously defined. Potential nucleic-acid targets include viruses, bacteria, protists, parasites, and antibiotic-resistance genes on plasmids. More speculatively, and dependent upon engineering beyond today’s norms, there is a possibility that viral nucleic acid in sewage is present inside host cells. Single cell isolation might allow identification of infected individuals directly from wastewater. This future technical capability raises questions concerning conflicts of privacy and public health [31].

The storage of samples for future nucleic acid analysis is currently not as widespread as its potential suggest it should be. What nudges [43] might encourage wider adaptation and what impediments might be ameliorated? At present the standards for sample preservation require either perpetual storage in the frozen state or the addition of nucleic acid stabilizing solutions that allow room temperature storage [25]. Room temperature storage volumes are large so that either method requires considerable resources. Purified nucleic acids are compact and stable for storage but processing samples is time-sensitive and resource demanding. Physical anthropological and forensic approaches have proven the ability to recover valuable sequence information from samples stored in a variety of ways and circumstances. There is a need for the convergence of approaches to sample archiving along with the appreciation of their future benefits and resources to apply them.

## Conclusions

Current sewage analysis includes some pharmaceuticals, cosmetics, illegal drugs, viruses, pesticides and other chemicals of concern [44–47]. The future is likely to see ever greater sophistication of analysis and expansion of the number of targets relevant to public health as well as increasing capacity for analysis as resources grow and/or become available with the passing of emergency situations. In anticipation, we encourage further sampling and preservation from built environments in order to prepare for a future that is able to efficiently utilize them [48]. Sample availability will enrich deeper historical understanding of what is unlikely to be the last pandemic [49]. Archiving samples now will enable the future’s understanding of its past and inform preparations for future challenges [50].

## Data Availability

Not applicable.
